# 542 Comparative Analysis of Unhoused vs. Housed Individuals in the Burn ICU

**DOI:** 10.1093/jbcr/irae036.176

**Published:** 2024-04-17

**Authors:** Paul Kantor

**Affiliations:** Torrance Memorial Medical Center, Redondo Beach, CA

## Abstract

**Introduction:**

Decreasing LOS by one day can decrease the cost of a hospital stay by 3% (Taheri et al., 2000). Several factors contribute to LOS in the BICU, including, but not limited to, age, comorbidities, acuity, treatment, exit strategy, and housing situation. The purpose of this study is to identify LOS differences between housing situations amongst BICU patients. Housing situations can delay or assist with LOS; unhoused individuals may experience a more complex exit strategy as compared with housed individuals, leading to greater LOS.

**Methods:**

A retrospective analysis utilizing the national burn database to gather information about discharged patients from TMMC BICU between 2018-2023. Factors such as age, gender, degree of burn, amount of burn (Total body surface area % [TBSA%]), and living situation were all considered.

Average LOS was calculated based on the patient’s documented housing situation. The total number of hospital days was divided by the number of patients in that category to calculate the average hospital days per patient. LOS Without Surgery population (n) total 475. LOS 2018-2023 n = 317.

**Results:**

A comparative analysis of Burn Registry data showed burn inpatients had longer LOSs compared to other inpatient burn groups LOSs of unhoused BICU inpatients averaged 2.8 days longer than housed patients.

**Conclusions:**

The cause of the prolonged LOS in unhoused populations is difficult to ascertain. Difficulties in arranging wound care and placement is a difficult task to complete by case management. Psychiatric and/or complicated medical histories contributed to delays in discharge, prompting opportunity for future research (Ikebuchi et al., 2008). Literature fails to provide consistent causative/correlational data for the length of stay amongst the burn population in the hospital setting. Current studies show that age, socioeconomic factors, comorbidities, antibiotic use, and burn severity/TBSA contribute to LOS in the burn population (Chukamei et al., 2021).

**Applicability of Research to Practice:**

Discharge planning should be initiated on the date of admission; special populations should be monitored more closely. Delays in discharge medication, wound care plans, and wound care supplies are intricate in these populations; research should be conducted into what causes these delays and what communication break downs between nursing, surgeons, and other ancillary staff prevent discharge. Involving a multidisciplinary approach would augment a safer and more expedited discharge for these complex patient populations. Future causative research is needed exploring reasons for discharge delays by examining interdisciplinary workflow communications that may contribute to delayed discharges.

